# Regional Brain Fusion: Graph Convolutional Network for Alzheimer's Disease Prediction and Analysis

**DOI:** 10.3389/fninf.2022.886365

**Published:** 2022-04-29

**Authors:** Wenchao Li, Jiaqi Zhao, Chenyu Shen, Jingwen Zhang, Ji Hu, Mang Xiao, Jiyong Zhang, Minghan Chen

**Affiliations:** ^1^Intelligent Information Processing Laboratory, Hangzhou Dianzi University, Hangzhou, China; ^2^Research Center for Healthcare Data Science, Zhejiang Lab, Hangzhou, China; ^3^Department of Computer Science, Wake Forest University, Winston-Salem, NC, United States; ^4^Sir Run Run Shaw Hospital, College of Medicine, Zhejiang University, Hangzhou, China

**Keywords:** brain network, amyloid-PET, regional brain fusion-graph convolutional network (RBF-GCN), adaptive native node attribute (ANNA), Alzheimer's disease

## Abstract

Alzheimer's disease (AD) has raised extensive concern in healthcare and academia as one of the most prevalent health threats to the elderly. Due to the irreversible nature of AD, early and accurate diagnoses are significant for effective prevention and treatment. However, diverse clinical symptoms and limited neuroimaging accuracy make diagnoses challenging. In this article, we built a brain network for each subject, which assembles several commonly used neuroimaging data simply and reasonably, including structural magnetic resonance imaging (MRI), diffusion-weighted imaging (DWI), and amyloid positron emission tomography (PET). Based on some existing research results, we applied statistical methods to analyze (i) the distinct affinity of AD burden on each brain region, (ii) the topological lateralization between left and right hemispheric sub-networks, and (iii) the asymmetry of the AD attacks on the left and right hemispheres. In the light of advances in graph convolutional networks for graph classifications and summarized characteristics of brain networks and AD pathologies, we proposed a regional brain fusion-graph convolutional network (RBF-GCN), which is constructed with an RBF framework mainly, including three sub-modules, namely, hemispheric network generation module, multichannel GCN module, and feature fusion module. In the multichannel GCN module, the improved GCN by our proposed adaptive native node attribute (ANNA) unit embeds within each channel independently. We not only fully verified the effectiveness of the RBF framework and ANNA unit but also achieved competitive results in multiple sets of AD stages' classification tasks using hundreds of experiments over the ADNI clinical dataset.

## Introduction

Alzheimer's disease (AD) is a progressive, degenerative, and irreversible brain disorder. Accurate early detections of AD offer enormous benefits to patients, families, and society as a whole (Alzheimer's Association, 2019). Due to advances in neuroimaging and machine learning technology in recent years, numerous machine learning algorithms have been developed to diagnose the stage of AD with various neuroimaging data and achieved some promising results (Klöppel et al., 2008; Vemuri et al., 2008; Cuingnet et al., 2011; Wee et al., 2011; O'Dwyer et al., 2012; Dyrba et al., 2013; Nir et al., 2015; Prasad et al., 2015; Zhang and Liu, 2018; Yan et al., 2019; Yang and Mohammed, 2020).

Compared with diffusion magnetic resonance imaging (dMRI), structural MRI (sMRI) is a more mature technology, making it more accessible in both clinical and academic settings. Various auxiliary diagnosis algorithms for AD have been developed based on sMRI data (Klöppel et al., 2008; Vemuri et al., 2008; Cuingnet et al., 2011; Tanveer et al., 2020), using the macrostructural changes of the brain, such as brain atrophy and neuronal tissue loss, to indicate AD progression. As dMRI techniques advance in recent years, some AD-related pathological studies have manifested that the microstructural changes of the brain may appear before macrostructural changes and occur in the early stages of the disease (Amlien and Fjell, 2014; Araque Caballero et al., 2018; Veale et al., 2021). Given the forward-looking nature of dMRI techniques in AD diagnosis, there have emerged many related discriminative method studies based on dMRI scans in recent years, which can be divided into two categories, namely, the diffusivity measures-based methods (O'Dwyer et al., 2012; Dyrba et al., 2013; Nir et al., 2015; Zhang and Liu, 2018) and network neuroscience-based methods (Wee et al., 2011; Prasad et al., 2015). Although the auxiliary diagnosis algorithm for AD based on dMRI scans has achieved good results, both types of methods mentioned above have their limitations. Among them, the former method usually only considers the local characteristics of nerve fibers. While the latter method can account for both local and global features to some extent, it will be inevitable to lose some key discriminative information when some specific network measures are selected artificially.

With the rise of graph convolutional networks (GCNs) for graph classification tasks on graph-structured data (Bruna et al., 2013; Henaff et al., 2015; Ying et al., 2018; Gao and Ji, 2019; Huang et al., 2019; Lee et al., 2019), a novel methodology is provided for an intelligent clinical diagnosis algorithm for AD based on network neuroscience. Song et al. (2019) directly utilized the conventional GCNs to discriminate the AD stages of subjects using the structural connectivity inputs derived from diffusion tensor imaging. However, although the conventional GCN can learn the potential representation information of graph-structured data more comprehensively, it cannot fully take the unique characteristics of AD pathology and the brain network into account. A closer topographical analysis reveals that the aggression of AD pathology exhibits different affinities for both hemispheres, which aligns with AD pathology studies (Giannakopoulos et al., 1994; Braak and Del Tredici, 2015; Ossenkoppele et al., 2016; Vogel et al., 2020). Meanwhile, some studies related to the brain network have proven the significant hemispheric lateralization of topology organization in the structural brain network (Iturria-Medina et al., 2011; Caeyenberghs and Leemans, 2014; Nusbaum et al., 2017; Yang et al., 2017). Some studies have also indicated that AD-related pathologies attack different brain regions in a certain sequence as the disease progresses (Crossley et al., 2014; Bischof et al., 2016; Cope et al., 2018; Pereira et al., 2019; Vogel et al., 2020).

Although Aβ status has been included in the revised diagnostic criteria for AD (Sperling et al., 2011), compared with other modalities of neuroimaging data, studies related to the AD diagnosis algorithms based on amyloid-PET data are still relatively rare (Vandenberghe et al., 2013; Yan et al., 2019). In the study by Son et al. (2020), the clinical feasibility of the deep learning method was validated in comparison with the visual rating or quantitative measures for evaluating the diagnosis and prognosis of subjects with equivocal amyloid-PET scans, so studies on an auxiliary diagnosis algorithm for AD based on amyloid-PET scans ought to receive more attention.

A large amount of literature demonstrates that data from multiple modalities, such as sMRI, DWI, fMRI, and PET biomarkers, can reveal the pathological characteristics of AD from different perspectives, so the fusion of complementary information from multimodal data can usually boost the performance of AD-related classifications and predictions. However, the conventional multimodality fusion methods, both direct (Schouten et al., 2016; Tang et al., 2016) and indirect (Yu et al., 2016; Zheng et al., 2017) mechanical combinations of multimodal features, neglect the intrinsic relationships between biomarkers derived from different modalities. For instance, some studies have revealed that amyloid-β (detected by PET) proteins spread along neural pathways (detected by dMRI) in a prion-like manner in AD progression (Iturria-Medina et al., 2014; Kim et al., 2019; Raj, 2021).

In this article, we proposed a novel regional brain fusion-graph convolutional network (RBF-GCN), which explicitly utilizes the characteristics of AD pathology and topological structure of brain networks simultaneously. To play the role of some more discriminative node attributes in AD diagnosis, we devised an adaptive native node attribute (ANNA) unit to improve the classic GCN, and the improved GCN can be embedded into the RBF framework. Inspired by the propagation mechanism of AD pathology and the working mechanism of GCNs based on message propagation, we used the graph-structured data to simply and naturally fuse the information from DWI scans (i.e., network topology) and amyloid-PET scans (i.e., nodal attributes).

The rest of this article is organized as follows. In the following sections, we describe the dataset used in this study as well as the proposed method. Later, we present details of our experimental results and discuss them. Finally, we conclude this article.

## Materials and Methods

### Data Description

Our dataset consists of 502 subjects, which includes 168 normal controls (NCs), 165 mild cognitive impairment (MCI), and 169 AD subjects. The demographic information of our dataset is shown in Table 1. All neuroimaging data of the subjects are selected from the Alzheimer's Disease Neuroimaging Initiative (ADNI; http://adni.loni.usc.edu), where each subject has scans of T1-weighted MRI, DWI, and amyloid-PET images. Amyloid-PET and T1-weighted MRI are jointly applied to calculate the regional amyloid standardized uptake value ratio (SUVR) level of each subject, and DWI and T1-weighted MRI are jointly used to construct the corresponding structural brain network. The related processing process of data is described in the next section.

**Table 1:** Demographic information.

**Diagnosis**	**Number**	**Age (year)**	**Gender (F/M)**	**Education (year)**	**MMSE**
NC	168	73.8 ± 5.5	91/77	16.8 ± 2.4	28.8 ± 1.4
MCI	165	72.0 ± 7.4	55/110	16.2 ± 2.7	27.7 ± 2.3
AD	169	74.2 ± 7.0	74/95	15.8 ± 2.7	24.7 ± 3.1

*Values are reported as mean ± SD. F/M, female/male; MMSE, Mini-Mental State Examination*.

### Data Processing

In this study, the Destrieux atlas (Destrieux et al., 2010) is used to calculate the amyloid SUVR of 148 cortical regions from the amyloid-PET scan and construct structural brain networks. Following the Destrieux atlas, the human cerebral cortex was parcellated into 74 different brain regions per hemisphere. The parcellation process of the cortical surface applied the standard internationally accepted nomenclature and criteria, so we selected this atlas. The data are processed using our in-house pipeline built on top of FreeSurfer (https://surfer.nmr.mgh.harvard.edu/) and FSL (FMRIB Software Library, https://fsl.fmrib.ox.ac.uk/fsl/fslwiki). Below is a detailed description of the processing steps.

For the SUVR calculation, we first applied a set of image processing steps on MR images to obtain the region parcellations. The preprocessing of each MR image consists of four major steps (Rajapakse et al., 1997; Tohka et al., 2004; Brendel et al., 2015), namely, (1) skull stripping; (2) tissue segmentation, where we segmented the volumetric intensity image into white matter (WM), gray matter (GM), and cerebrospinal fluid (CSF); (3) constructing the cortical surface based on tissue segmentation results; and (4) registering the Destrieux atlas to the underlying MR image using deformable image registration, which allows us to map the region parcellation from atlas space to individual space. We then performed an image registration between amyloid-PET images and associated MR images such that two imaging modality data are spatially aligned. Next, we selected the cerebellum as the reference region to calculate the SUVR for each brain region, which is essentially equal to the ratio between the average SUV in the region under consideration and the average SUV at the cerebellum (Vogel et al., 2020; Gonzalez-Escamilla et al., 2021). To construct the structural brain network, based on the Destrieux parcellation of the MR image, we used a seed-based probabilistic fiber tractography method over the DWI scan data, which determines how many fibers (directly) connect any two brain regions (Messaritaki et al., 2019). Then, a structural brain network consisting of 148 nodes and associated edges is constructed where connectivity strength reflects the number of fibers between two nodes.

### Problem Statement

**Figure 1:**
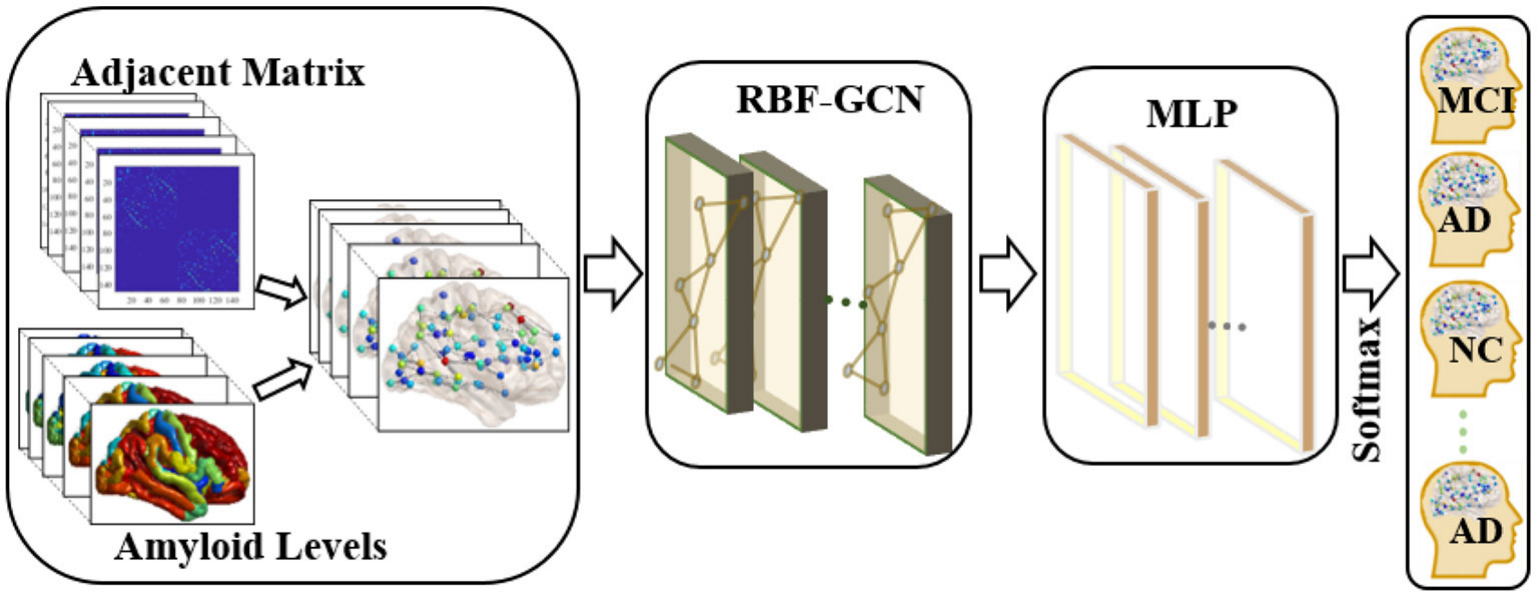
Overview of the pipeline used in the classification of each subject's clinical diagnosis. First, we applied the graph-structured data to incorporate the topology of the brain network and nodal attributes (i.e., amyloid levels) in each brain region. Then, a regional brain fusion-graph convolutional network (RBF-GCN) model is developed to extract the underlying features of the graph-structured data. Finally, a multilayer perceptron (MLP) module and a Softmax layer are added to predict each subject's Alzheimer's disease (AD) stage.

In this study, subjects *S* = [*s*_1_, ⋯  , *s*_*n*_] associate with their structural brain network ℕ = {*V, L, F*}, where *V* denotes nodes (brain regions) set, *L* represents links (white matter fiber bundles between every two nodes) set, and *F* denotes the node attribute matrix. More specifically, *m* = |*V*| represents the total number of nodes in each brain network, and each node *v*_*i*_ ∈ *V* has a *d*-dimensional attribute feature representation denoted by *f*_*i*_, which could naturally incorporate the nodal identification code, amyloid level, and so on. Hence, a subject's node attribute matrix is $F=\left[f_{1};\cdots \;;f_{m}\right]\in R^{m\times d}$. The weighted adjacent matrix *A* ∈ *R*^*m*×*m*^ quantitatively describes the strength of network links *L*. *Y* = [*y*_1_, ⋯  , *y*_*n*_] represents the status of subjects, and a subject's status *y*_*i*_ belongs to one of the three diagnostic labels, namely, NC, MCI, and AD. As illustrated in Figure 1, our objective is to discriminate each subject's status using the model learned from the aforementioned brain network dataset.

### Graph Convolutional Network

Based on the information propagation mechanism, the classical GCN can be used to integrate the structural information of the brain network into the nodal feature representation, which is commonly defined as (Kipf and Max, 2017; Huang et al., 2019; Ranjan et al., 2020):


(1) $$\begin{array}{l} \begin{array}{c} H^{\left(k+1\right)}=\sigma \left({\overset{\;}{D}}^{-\frac{1}{2}}\overset{\;}{A}{\overset{\;}{D}}^{-\frac{1}{2}}H^{\left(k\right)}W^{\left(k\right)}\right), \end{array} \end{array}$$

*H*^(*k*)^denotes the input of the *k*-th GCN layer, and *H*^(0)^is initialized with the initial node attribute matrix, i.e., *H*^(0)^ = *F*. $\overset{\;}{A}=A+I_{m}$ represents the adjacent matrix of the network with self-loop, and *I*_*m*_ denotes the identity matrix. The diagonal matrix $\overset{\;}{D}$ is computed by ${\overset{\;}{D}}_{ii}=\underset{j}{\sum }{\overset{\;}{A}}_{ij}$ and ${\overset{\;}{D}}_{ij}=0$ for *i* ≠ *j*. *W*^(*k*)^ ∈ *R*^*d*^(*k*)^×*d*^(*k*+1)^^ is a learnable parameter matrix in the *k*-th GCN layer, which is shared by each node of the brain network.

### Regional Brain Fusion-Graph Convolutional Network

**Figure 2:**
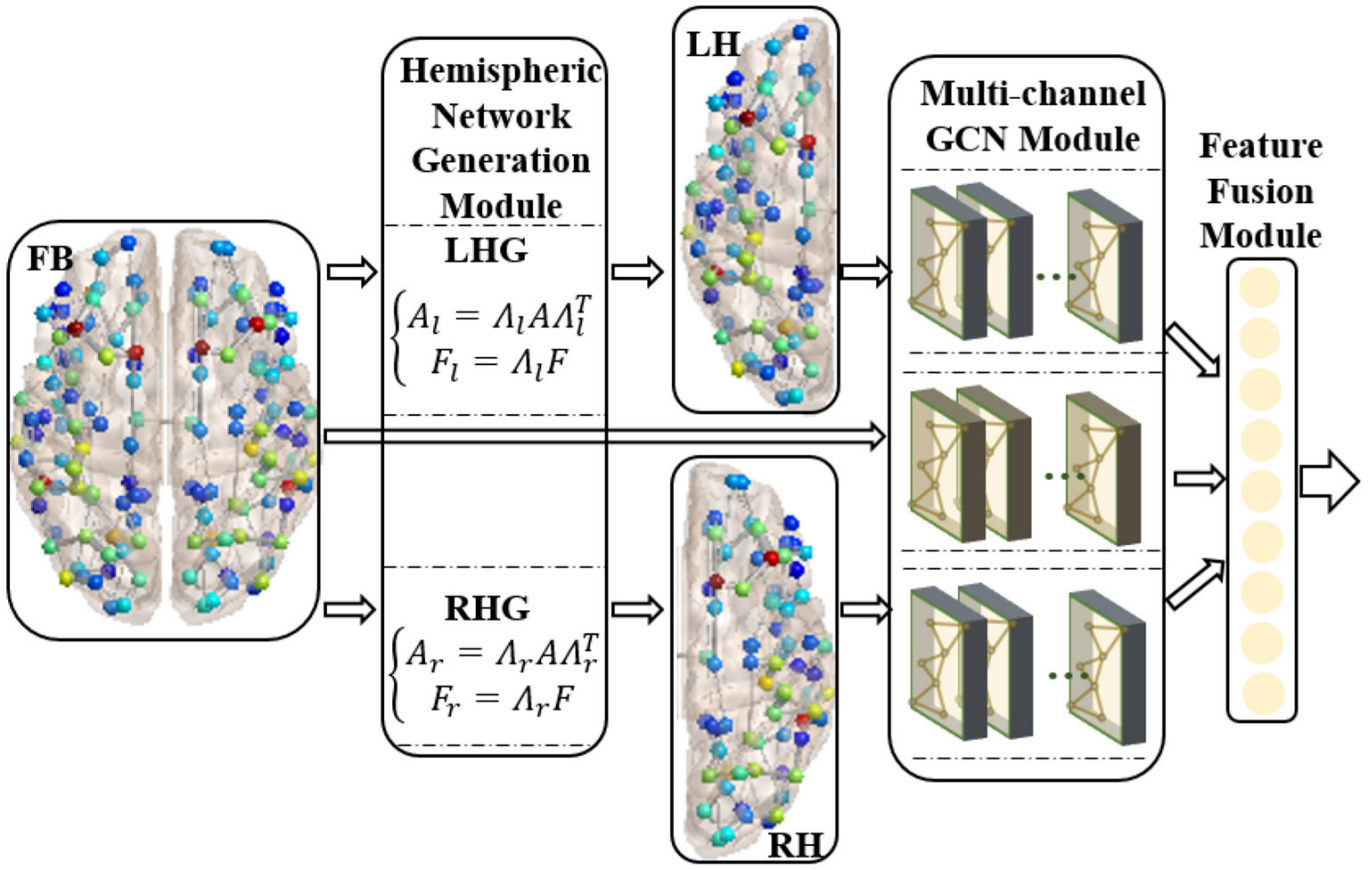
Regional brain fusion framework. FB, LH, and RH denote full brain network and left and right hemispheric subnetwork, respectively. LHG and RHG separately denote left and right hemispheric network generation modules. FB and LH generated by LHG and RH generated by RHG are input to the corresponding channels in the multichannel GCN module, and then the extracted representation information is merged in the feature fusion module.

We proposed the RBF-GCN inspired by the asymmetry of AD pathology between hemispheres (Giannakopoulos et al., 1994; Braak and Del Tredici, 2015; Ossenkoppele et al., 2016; Vogel et al., 2020) and the hemispheric lateralization of topology organization in the structural brain network (Iturria-Medina et al., 2011; Caeyenberghs and Leemans, 2014; Nusbaum et al., 2017; Yang et al., 2017). As illustrated in Figure 2, RBF-GCN is built using an RBF framework mainly comprised of three modules, namely, (1) hemispheric network generation module, which generates left and right hemispheric subnetwork based on the whole brain network, (2) multichannel GCN module, which extracts the representation information from the left and right hemispheric and the full brain, respectively, and (3) feature fusion module (Lu et al., 2020), which merges feature vectors from left and right hemispheres and the full brain. In the multichannel GCN module of RBF-GCN, the improved GCN enhanced by our proposed ANNA unit is embedded into each channel independently.

#### Hemispheric Network Generation Module

The relative subnetworks of both hemispheres are generated by the following operation:


(2) $$\begin{array}{l} \{\begin{array}{c} A_{l}=\Lambda_{l}A\Lambda_{l}^{T},A_{l}\in R^{\left(\frac{m}{2}\right)\times \left(\frac{m}{2}\right)}\\ A_{r}=\Lambda_{r}A\Lambda_{r}^{T},A_{r}\in R^{\left(\frac{m}{2}\right)\times \left(\frac{m}{2}\right)}\\ F_{l}=\Lambda_{l}F,F_{l}\in R^{\left(\frac{m}{2}\right)\times d}\\ F_{r}=\Lambda_{r}F,F_{r}\in R^{\left(\frac{m}{2}\right)\times d} \end{array} \end{array}$$

where *A*_*l*_, *A*_*r*_ and *F*_*l*_, *F*_*r*_ denote the weighted adjacent matrix and node attribute matrix of left/right hemispheric networks, respectively (the left and right hemispheric networks have the same number of nodes). Λ_*l*_ and Λ_*r*_ are used to extract the left and right hemispheric subnetworks and node attribute matrices. When corresponding nodes are arranged in the order to construct the adjacency matrix of the whole brain network, the above two matrices are defined as follows:


(3) $$\begin{array}{l} \{\begin{array}{c} {\left(\Lambda_{l}\right)}_{ij}=\{\begin{array}{c} 1\;j=i\\ 0\;others \end{array},\Lambda_{l}\in R^{\left(\frac{m}{2}\right)\times m}\\ {\left(\Lambda_{r}\right)}_{ij}=\{\begin{array}{c} 1\;j=i+\left(\frac{m}{2}\right)\\ 0\;others \end{array},\Lambda_{r}\in R^{\left(\frac{m}{2}\right)\times m} \end{array} \end{array}$$

#### Adaptive Native Node Attribute Unit

**Figure 3:**
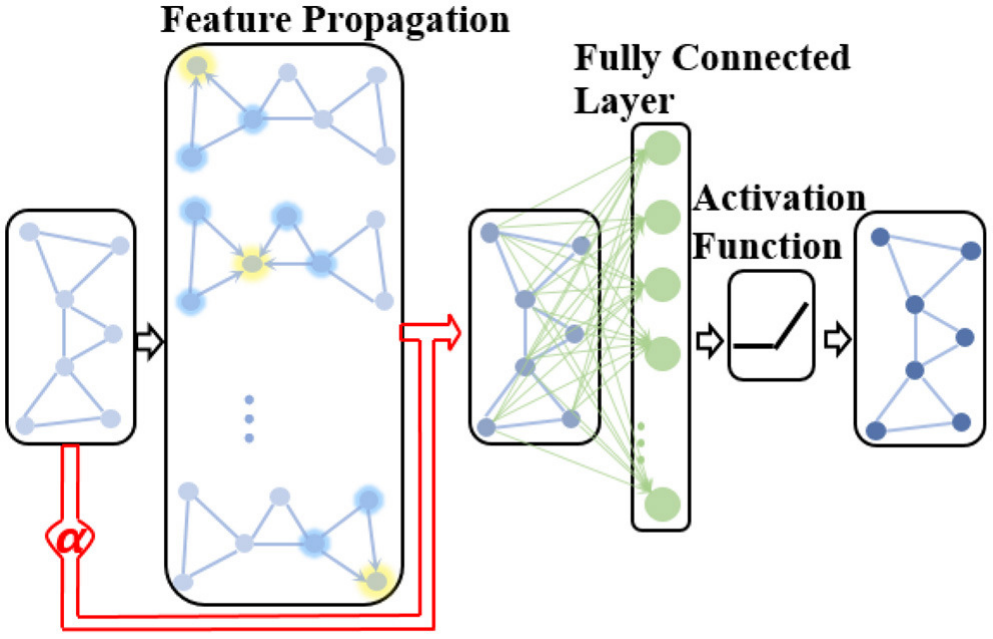
The structure of the improved GCN with the adaptive native node attribute (ANNA) unit. The red section indicates the ANNA unit, which adaptively adjusts the contribution of native node features in the extracted network representation.

In a classical GCN, the increased depth of GCN allows each node to aggregate features from more topologically distant k-hop nodes, which could encode structural information of brain networks more comprehensively. Meanwhile, the attribute features representation at each node is smoothed. However, a consensus has emerged that AD attacks different brain regions in a certain sequence during disease progression in various existing AD pathological studies (Crossley et al., 2014; Bischof et al., 2016; Cope et al., 2018; Pereira et al., 2019; Vogel et al., 2020). From this, it can be inferred that the expressive power of attribute features of some specific brain network nodes (brain regions) is relatively stronger, which is more conducive to AD diagnosis. To evaluate the importance of regional structural network and attribute features in determining AD stages, we improved the classic GCN by adding an ANNA unit, which is more suitable for brain network data. The improved GCN is defined as follows:


(4) $$\begin{array}{l} \begin{array}{c} H^{\left(k+1\right)}=\sigma \left(\left({\overset{\;}{D}}^{-\frac{1}{2}}\overset{\;}{A}{\overset{\;}{D}}^{-\frac{1}{2}}H^{\left(k\right)}+\alpha^{\left(k\right)}H^{\left(k\right)}\right)W^{\left(k\right)}\right), \end{array} \end{array}$$

where α^(*k*)^ is a learnable parameter in the *k*-th GCN layer used to adaptively adjust the contribution of the native node attribute in the extracted network representation. Therefore, the proposed ANNA unit can play the role of multimodal data simultaneously when diagnosing the AD stages, thereby improving the prediction accuracy. The overall structure of the improved GCN is shown in Figure 3.

#### Feature Fusion Module

To improve the discrimination capacity of the feature maps of the brain network, we explored two commonly used feature fusion methods, namely, concatenation and addition, to integrate the full brain network and left and right hemispheric subnetworks (Ma et al., 2019; Du et al., 2020; Saad et al., 2022).

The process of concatenation can be described by the following formula:


(5) $$\begin{array}{l} \begin{array}{c} H_{concat}=\left[H_{f},H_{l},H_{r}\right], \end{array} \end{array}$$

where *H*_*f*_, *H*_*l*_, and *H*_*r*_ denote the feature maps of full brain network and left and right hemispheric subnetworks, which are output by the corresponding channels of the multichannel GCN module. The combined feature map is denoted by *H*_*concat*_, whose column dimension size is equal to the sum of the corresponding values of the right three feature maps. So, the feature map generated by this method owns the more diverse feature representation.

The process of addition is performed as the following formula:


(6) $$\begin{array}{l} \begin{array}{c} H_{add}=H_{f}⊕H_{l}⊕H_{r}, \end{array} \end{array}$$

where ⊕ means element-wise addition. The three items on the right side of the formula must have the same shape (i.e., both row and column dimensions must match), and their sum, the combined feature map *H*_*add*_, also shares the same shape right-hand side. Compared with concatenation, addition is more space efficient: this fusion method condenses the feature representation information in a relatively narrower dimensional space, which puts forward higher requirements for the classifier's ability to identify feature details.

## Experimental Setup

To evaluate the performance of our proposed method, we implemented three binary classification tasks (i.e., NC vs. AD, NC vs. MCI, and MCI vs. AD) and one multiclass classification task (i.e., NC vs. MCI vs. AD) on the real-world dataset from the ADNI database in AD diagnosis. In recent years, although computer-aided diagnosis research has achieved breakthrough results in the first binary classification task (i.e., NC vs. AD), the remaining three classification tasks have relatively more room for improvement (Tanveer et al., 2020). Thus, for more refined disease state transformation prediction and more accurate early screening, we focused more on the remaining three classification tasks.

All our experiments employ 10-fold cross-validation to ensure a fair performance evaluation. A comprehensive evaluation of the classifier is conducted by simultaneously examining three quantitative aspects, namely, accuracy (ACC), sensitivity (SEN), and specificity (SPE) (Liu et al., 2016). As an exception, multiclass classification only checks accuracy. Following are the formulas for calculating the three metrics (Baratloo et al., 2015):


(7) $$\begin{array}{l} \text{ACC}=\;\frac{TP+TN}{TP+FN+TN+FP}, \end{array}$$

(8) $$\begin{array}{l} \text{SEN}=\;\frac{TP}{TP+FN}, \end{array}$$

(9) $$\begin{array}{l} \text{SPE}=\;\frac{TN}{TN+FP}, \end{array}$$

where TP, FP, TN, and FN represent the numbers of subjects correctly identified as patients, incorrectly identified as patients, correctly identified as healthy, and incorrectly identified as healthy, respectively. For each classification task, a group of subjects with relatively serious illnesses is considered patients, while the rest are considered healthy, such as classification task (i.e., MCI vs. AD), AD subjects are considered patients, and MCI subjects are considered healthy. We established a 3-layered GCN with 32 hidden units as the baseline in our experiments. For the fairness of comparison, our RBF-GCN model also adopts a 3-layered network, and all three network channels of the whole brain, and each hemisphere owns 32 hidden units (Huang et al., 2019). The MLP modules used in our experiments are all 2-layered fully connected networks composed of 32 hidden units. In the model training session, the epoch size is set to 500, the learning rate is set to 0.001, the batch size is set to 20, the weight parameters are initialized using the Xavier normal distribution (Glorot and Bengio, 2010), and the negative log-likelihood loss function and Adam method (Kingma and Ba, 2014) are applied to optimize the model.

## Results

This section first verifies the effectiveness of our ANNA unit and RBF framework through ablation experiments and then verifies the superior performance of our proposed RBF-GCN model on multiple sets of AD stages classification tasks through comparative experiments.

### Effect of ANNA Unit

**Figure 4:**
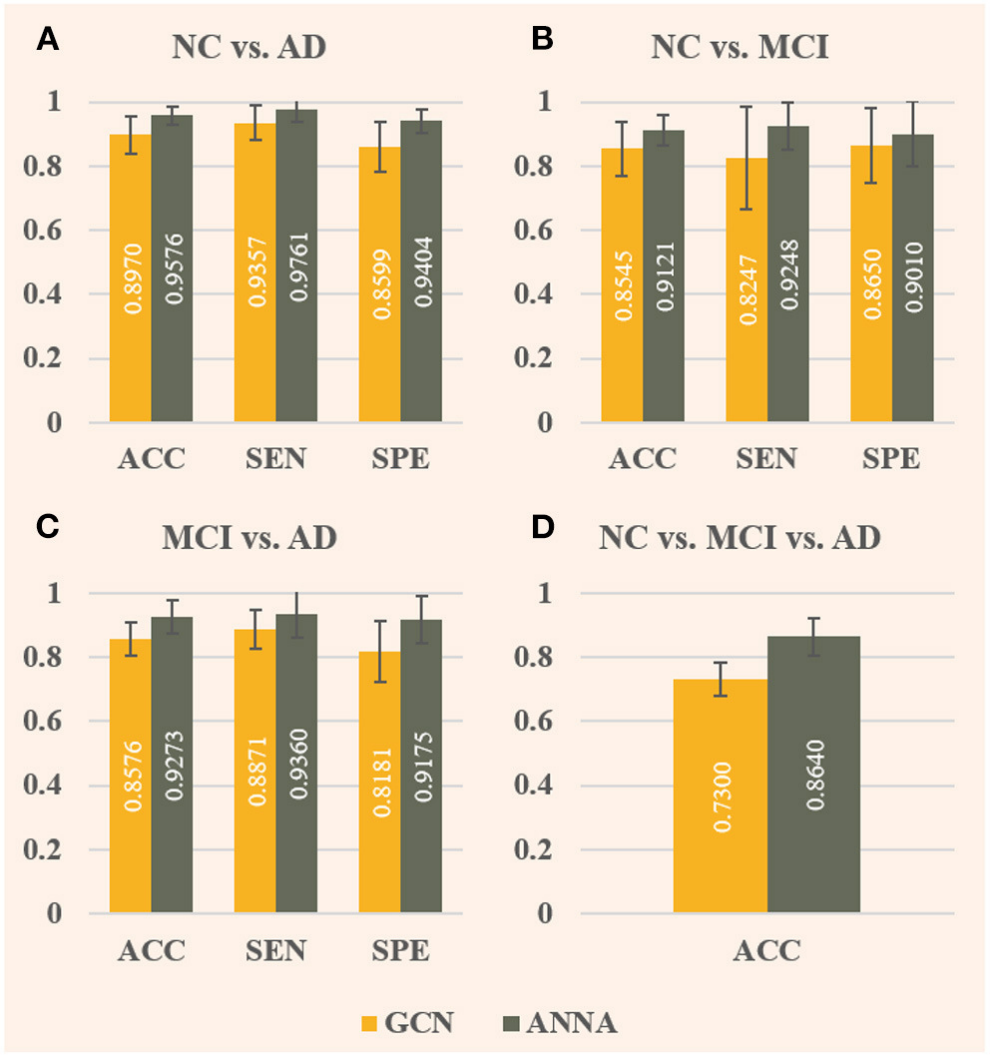
Evaluation of baseline GCN and the improved GCN with ANNA unit for AD stages classification. The baseline GCN is denoted by GCN and plotted in yellow; the GCN with ANNA unit is denoted by ANNA and plotted in green. The specific index for each column is displayed in white in the middle. The error bars depict the standard deviation of the three evaluation indicators across the 10 different folds, respectively. The panels **(A–D)**, respectively, display the results of the four groups of classification tasks.

As shown in Figure 4, the ANNA unit can effectively improve the classification performance on all four tasks. For the fairness of comparison, both models employed the same parameter configuration and training method except for the ANNA unit. Regarding classification accuracy, the highest improvement was obtained on the multiclassification task shown in Figure 4D, which increased by 18.36%, and the classification task with the smallest improvement (i.e., NC vs. MCI) also increased by 6.74%, which is only slightly lower than 6.76% from the classification task (i.e., NC vs. AD). With regards to sensitivity, the tasks with greater difficulty of discrimination (i.e., NC vs. MCI and MCI vs. AD) show a relatively higher improvement, which is 12.14 and 5.51%, respectively. Therefore, it is reasonable to deduce that the improved GCN enhanced by the ANNA unit has a more pronounced effect on more difficult classification tasks. As for specificity, the highest improvement was on the task MCI vs. AD, which increased by 12.15%; the smallest improvement was on the task NC vs. MCI, which increased by 4.16%. In addition, as the error bars demonstrate, the GCN with the ANNA unit generally has a smaller standard deviation in performance, which indicates that the training of this model is more stable.

### Effect of RBF Framework

**Figure 5:**
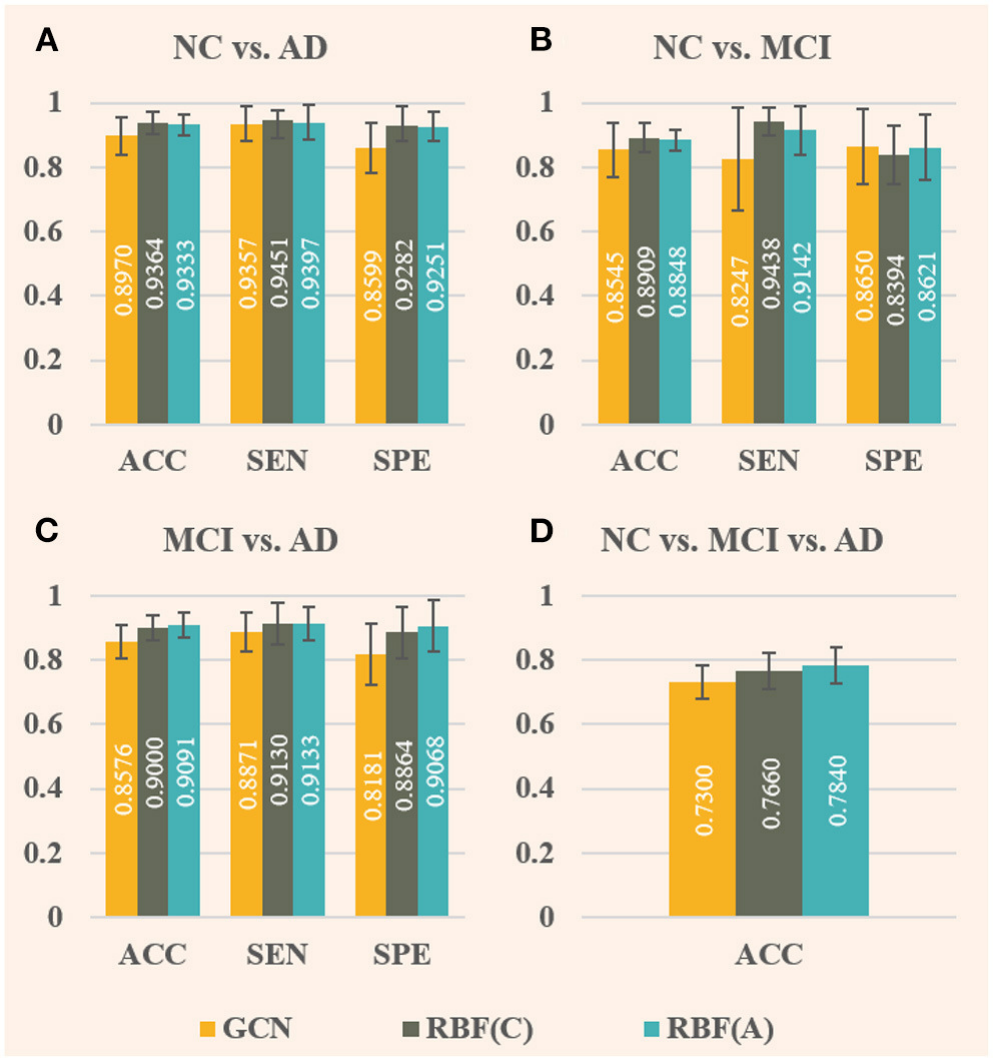
Comparison of baseline GCN (denoted by GCN, in yellow) and both improved GCNs with RBF framework [denoted by RBF **(C)**, in green, and denoted by RBF **(A)**, in blue] for AD stages classification. Among them, RBF **(C)** corresponds to the RBF framework using the concatenation module, whereas RBF **(A)** corresponds to the one using the addition module. In this case, only baseline GCNs are embedded in the multichannel GCN module of the RBF framework. The specific index for each column is displayed in white in the middle. The error bars depict the standard deviation of the three evaluation indicators across the 10-folds, respectively. The panels **(A–D)**, respectively, display the results of the four groups of classification tasks.

As illustrated in Figure 5, the effect of RBF framework is compared with the baseline GCN (denoted by GCN), where RBF (C) denotes RBF framework with the concatenation module and RBF (A) denotes the one with the addition module. Comparing Figures 4, 5, the overall performance improvement of the RBF framework is not as significant as that of the ANNA unit, however, which can also improve the classification performance on all four tasks quite well. Regarding classification accuracy, RBF (C) performs better in the first two tasks (i.e., NC vs. AD and NC vs. MCI), that is, the accuracy improvement is more pronounced. Contrariwise, RBF (A) performs better in the other two tasks. Among them, the highest accuracy improvement (7.40%) is achieved by RBF (A) in the multiclassification task, and the lowest one (3.55%) appears in the task NC vs. MCI, which is also obtained by RBF (A). In contrast, RBF (A) is more dependent on data, whereas a task-balanced RBF (C) facilitates the promotion. With regards to sensitivity, both RBF (C) and RBF (A) performed exceptionally well with tasks NC vs. MCI, the former improving by up to 14.44%, but their corresponding specificity manifested slightly negative growth. Methods that compromise sensitivity and specificity can be more clinically accepted. For the other two binary classification tasks, in terms of specificity, both RBF (C) and RBF (A) achieved quite high-performance improvements. Among them, the highest (10.84%) and the lowest (7.58%) were obtained by RBF (A) in the task MCI vs. AD and task NC vs. AD, respectively. As illustrated by the error bars of Figure 5, the RBF framework can also enhance the stability of the model on the test set.

### Comparison With Existing Methods

Besides investigating how the different components of our proposed RBF-GCN model impact AD stages classification performance, we further compared the performance of RBF-GCN to several competitive methods proposed in recent literature, which include (1) Multiscale Laplacian Graph (MLG) kernel, which is a multilevel, recursively defined kernel that captures topological relationships between individual vertices, and between sub-graphs (Kondor and Pan, 2016); (2) graph2vec, which defines an unsupervised representation learning method to learn the embedding of graphs of arbitrary sizes (Narayanan et al., 2017); (3) Infograph, which is an unsupervised graph representation learning method that applies contrastive learning to graph learning by maximizing the mutual information between both graph-level and node-level representations (Sun et al., 2019); (4) PSCN (PATCHY-SAN), which extracts and normalizes a neighborhood of exactly k nodes for every node and then uses selected neighborhood as the receptive field in traditional convolutional operations (Niepert et al., 2016); and (5) baseline GCN. To reduce the impact of external factors, such as model training and evaluation process on evaluation fairness, we implemented the aforementioned methods using libraries GraKeL (Siglidis et al., 2020) and CogDL (Cen et al., 2021) in Python and Pytorch. Some of the hyperparameters were adjusted to achieve the best performance. These models were trained and evaluated on our dataset of 502 subjects with 10-fold cross-validation.

As shown in Table 2, when compared with the first four typical graph classification methods, baseline GCN model achieves promising performance, especially for the task NC vs. MCI vs. AD: the classification accuracies for the first four methods are about 50%, while baseline GCN model achieves 73%. This result once again confirms that GCN is a powerful and promising tool in AD stage classification studies. Given the outstanding performance of baseline GCN, we directly used it as a benchmark and compared the proposed RBF-GCN (C) and RBF-GCN (A) models with it. For the task NC vs. AD, in terms of accuracy, sensitivity, and specificity, RBF-GCN (C) and RBF-GCN (A) increased by 7.09, 4.32, 10.08% and 7.09, 5.58, 8.63%, respectively. Both models achieved the same classification accuracy (96.06%). For the task NC vs. MCI, in terms of accuracy, sensitivity, and specificity, RBF-GCN (C) and RBF-GCN (A) increased by 7.81, 14.90, 4.54% and 8.52, 13.46, 6.94%, respectively. Compared with the results of GCN with ANNA unit and RBF framework, RBF-GCN (C) and RBF-GCN (A) noticeably improve accuracy and specificity. For the task MCI vs. AD, in terms of accuracy, sensitivity, and specificity, RBF-GCN (C) and RBF-GCN (A) increased by 10.95, 11.27, 11.72 and 10.95, 9.45, 14.46, respectively. Similar to their performance on the task NC vs. AD, both methods achieved the same accuracy (95.15%). For the task NC vs. MCI vs. AD, RBF-GCN (C) and RBF-GCN (A) inherited the superior performance of classic GCN and achieved an accuracy of 90 and 87.2%, respectively, an increase of 23.29 and 19.45%. For all four tasks, RBF-GCN (A) and RBF-GCN (C) achieved the lowest standard deviations of the evaluation metrics. In summary, both methods achieved better performance on multiple tasks than the GCN with the ANNA unit and RBF framework. Their performance appears to be closer, implying that the ANNA unit improves the adaptability of the RBF framework to a certain extent and has the potential for clinical applications. With the same preconditions, compared with the baseline GCN, our proposed RBF-GCN model has significantly improved the classification performance. Although the computational cost has increased slightly, the network size is relatively small, making it still quite competitive in terms of computational cost. Each epoch takes about 0.6 s on a machine with an NVIDIA GeForce GTX 1080 Ti GPU (11 GB memory), 8-core Intel Xeon CPU (3.0 GHz), and 64 GB of RAM.

**Table 2:** Comparison of the classification performances.

**Task**	**Method**	**ACC(%)**	**SEN(%)**	**SPE(%)**
NC vs. AD	MLG	73.07 ± 4.75	70.73 ± 9.51	75.41 ± 7.75
	graph2vec	70.91 ± 7.76	71.35 ± 8.95	70.38 ± 11.07
	Infograph	70.67 ± 5.02	63.40 ± 10.29	77.73 ± 7.67
	PSCN	74.20 ± 9.08	69.71 ± 11.45	78.64 ± 9.18
	GCN	89.70 ± 5.94	93.57 ± 5.61	85.99 ± 7.95
	RBF-GCN(C)	**96.06** **±3.60**	97.61 ± 3.94	**94.66** **±4.15**
	RBF-GCN(A)	**96.06** **±3.60**	**98.79** **±2.43**	93.41 ± 2.43
NC vs. MCI	MLG	65.15 ± 8.27	65.94 ± 11.17	63.65 ± 13.63
	graph2vec	66.36 ± 8.07	60.42 ± 5.34	71.81 ± 18.04
	Infograph	56.67 ± 10.41	41.56 ± 15.46	72.27 ± 15.74
	PSCN	61.82 ± 8.15	48.60 ± 19.47	74.49 ± 11.94
	GCN	85.45 ± 8.44	82.47 ± 15.96	86.50 ± 11.66
	RBF-GCN(C)	92.12 ± 2.78	**94.76** **±4.70**	90.43 ± 6.53
	RBF-GCN(A)	**92.73** **±2.78**	93.57 ± 7.67	**92.50** **±7.49**
MCI vs. AD	MLG	68.58 ± 9.05	65.36 ± 11.69	72.31 ± 14.00
	graph2vec	65.89 ± 6.48	68.70 ± 7.93	64.80 ± 9.82
	Infograph	65.86 ± 9.72	59.85 ± 10.64	72.18 ± 11.97
	PSCN	64.32 ± 8.51	62.46 ± 15.23	65.96 ± 20.27
	GCN	85.76 ± 5.26	88.71 ± 5.95	81.81 ± 9.52
	RBF-GCN(C)	**95.15** **±3.37**	**98.71** **±2.59**	91.40 ± 7.19
	RBF-GCN(A)	**95.15** **±3.64**	97.09 ± 5.22	**93.64** **±6.80**
NC vs. MCI vs. AD	MLG	54.05 ± 6.70	~	~
	graph2vec	52.43 ± 6.82	~	~
	Infograph	51.64 ± 8.01	~	~
	PSCN	50.82 ± 4.17	~	~
	GCN	73.00 ± 5.16	~	~
	RBF-GCN(C)	**90.00** **±5.51**	~	~
	RBF-GCN(A)	87.20 ± 4.21	~	

*The bold values represent the best results for a single evaluation metric when multiple methods are used in the same classification task*.

## Discussion

In this section, we examine the pathological basis for designing the ANNA unit and RBF framework with statistical analysis.

### Pathological Basis of ANNA Unit

**Figure 6:**
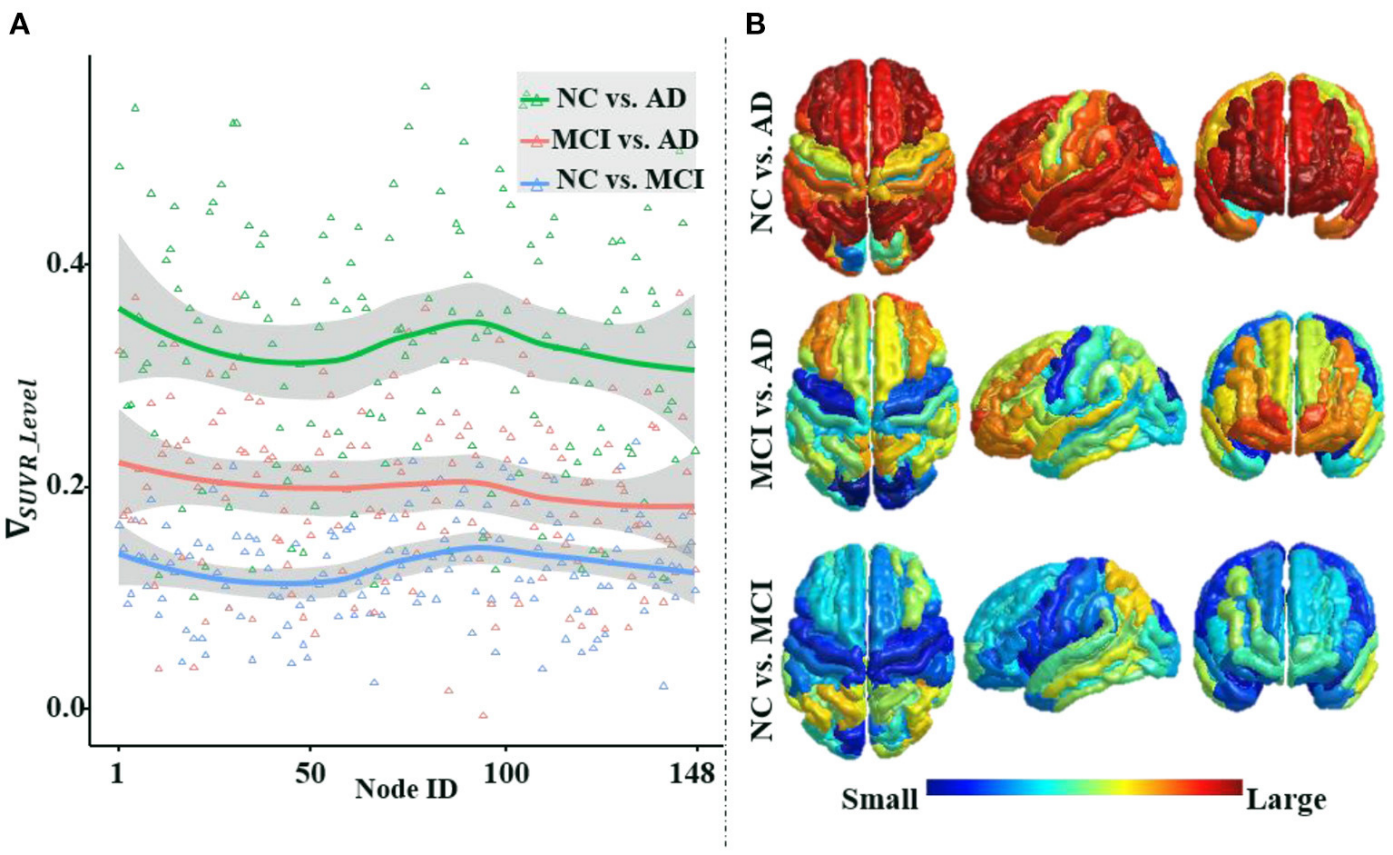
An illustration of the differences in the average regional amyloid level between cohorts. The three curves from top to bottom in panle **(A)**, respectively, depict the differences in the average amyloid level of each brain region between cohorts NC vs. AD, MCI vs. AD, and NC vs. MCI. Corresponding to panel **(A)**, from top to bottom, panel **(B)** depicts the brain region maps of the average amyloid level differences between the cohorts. Node IDs (1–74) correspond to the 74 brain regions in the left hemisphere, node IDs (75–148) to the 74 brain regions in the right hemisphere, and within each hemisphere, the brain regions are numbered based on Destrieux atlas parcellation. MCI, mild cognitive impairment; NC, normal control.

Figure 6 illustrates that the differences in average amyloid levels between cohorts vary by brain region, implying that AD burdens have different affinities for different brain regions, which are consistent with the results of numerous existing studies (Crossley et al., 2014; Bischof et al., 2016; Cope et al., 2018; Pereira et al., 2019; Vogel et al., 2020). This further validates the necessity of our proposed ANNA unit, which is conducive to playing the role of brain region attributes with more obvious discrimination in AD diagnosis. Through horizontal comparison across the three cohort pairs (i.e., NC vs. AD, MCI vs. AD, and NC vs. MCI), it is clear that the magnitude of the differences in amyloid levels is from large to small in order. Among them, the difference in the amyloid level from the cohort pair (i.e., NC vs. AD) is significantly larger than the other two cohort pairs, and the gap between the other two cohort pairs (i.e., MCI vs. AD and NC vs. MCI) is not large. This is not only consistent with the general clinical manifestations of AD development but also consistent with our experimental results, that is, the performance of the same classification method on the task NC vs. AD is significantly better than the other two binary classification tasks. In sum, the mutual confirmation between the manifestation of amyloid-β in AD pathological development and our experimental results more comprehensively substantiates the necessity and effectiveness of our proposed ANNA unit.

### Pathological Basis of RBF Framework

**Figure 7:**
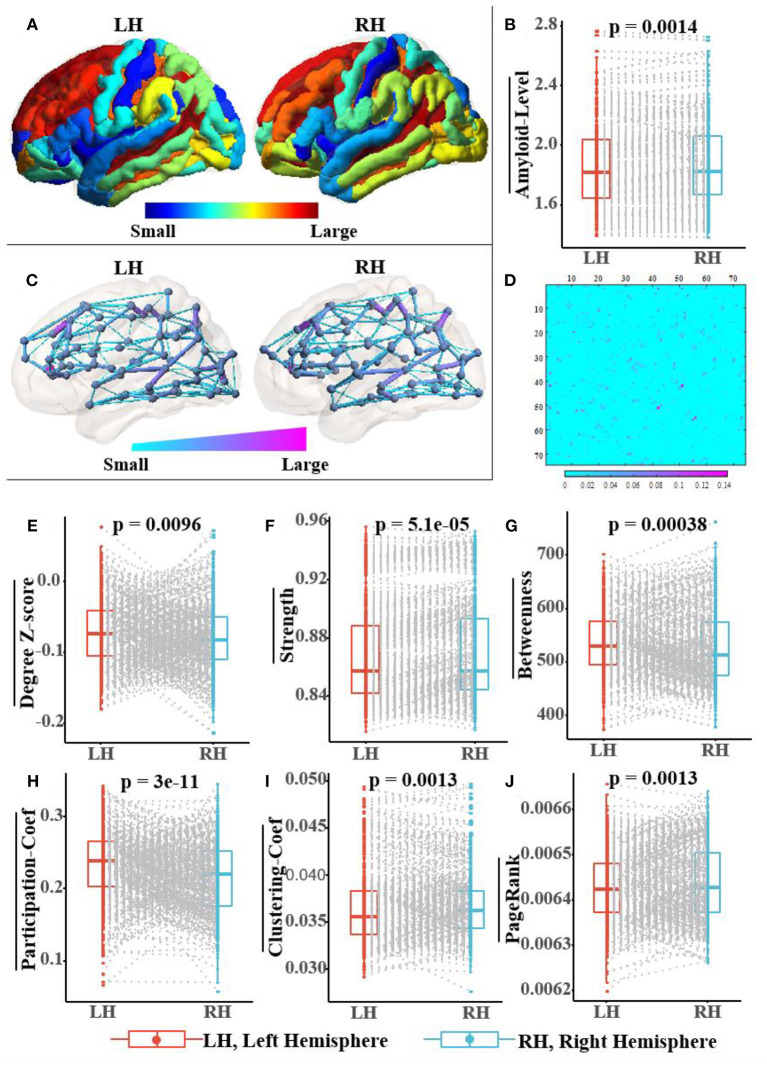
Population-wide comparison between left and right hemispheres. Panel **(A)** exhibits the average amyloid level map of the corresponding brain regions on the left and right hemispheres. Panel **(B)** shows the paired comparison box plots of the average amyloid levels for the respective brain regions in the left and right hemispheres. Panel **(C)** exhibits the population topological structures of the left and right hemispheres. Panel **(D)** displays the difference matrix of the adjacency matrix of the left and right hemispheres. Panel **(E–J)** show the paired comparison box plots of the six graph measures of the brain networks from the left and right hemispheres, respectively. Pairs of brain regions in the left and right hemispheres with the same name are matched in the paired samples Wilcoxon test.

Figure 7 not only shows a significant difference in the distribution of amyloid-β between left and right hemispheres but also in the topological structure of the subnetworks of both hemispheres. In Figure 7A, we visualized the asymmetric distribution of amyloid-β in the left and right hemispheres and then further check it with a Wilcoxon signed-rank test in Figure 7B, which is consistent with existing research results on AD pathology (Giannakopoulos et al., 1994; Braak and Del Tredici, 2015; Ossenkoppele et al., 2016; Vogel et al., 2020). As reported in some literature (Iturria-Medina et al., 2011; Caeyenberghs and Leemans, 2014; Nusbaum et al., 2017; Yang et al., 2017), Figures 7C,D visualize the hemispheric lateralization of topology organization in the structural brain network, which further illustrates with six typical graph measures, including the within-module degree z-score, strength, betweenness, participation coefficient, clustering coefficient, and Pagerank (Kaiser, 2011; Fornito et al., 2016). Among them, two measurements (i.e., the within-module degree z-score and participation coefficient) reveal the modularity of the brain network, while another three centrality measurements (i.e., strength, Pagerank, and betweenness) and clustering coefficient jointly reveal the hierarchical nature of the brain network. In Figures 7E–J, the Wilcoxon signed-rank test indicates the significant hemispheric lateralization of the brain network. Briefly, the above statistical analysis results firmly prove the rationality of our RBF framework design concept; at the same time, the improved experimental results can validate the analysis results in turn.

## Conclusion

In this study, we introduced RBF-GCN, a novel model specifically for AD stages classification based on graph-level classification. An RBF framework is devised to explicitly exploit the asymmetry of AD pathology and the lateralization of the brain network between both hemispheres. Meanwhile, an ANNA unit is proposed to adaptively assess the role of the structural information of the brain network and the attribute feature information at each node. We validated the effectiveness of the RBF-GCN components using ablation experiments and data-driven statistical analysis. Through extensive experiments, we demonstrated that RBF-GCN reaches state-of-the-art performance on AD stages classification tasks. Future research should explore the possibility of integrating different GCNs with higher expressive power into the multichannel GCN module to further improve the performance of RBF-GCN. Regarding multimodality fusion, more biomarker information about AD pathology should be incorporated to improve the accuracy of the diagnosis algorithm. Additionally, larger-scale datasets should be constructed to verify the generalization ability of the model.

## Data Availability Statement

The original contributions presented in the study are included in the article/supplementary material, further inquiries can be directed to the corresponding author/s.

## Author Contributions

WL contributed to conceptualization, methodology, software, data curation, and writing. JiaZ and CS contributed to methodology and software. JinZ contributed to writing the manuscript. JH and MX contributed to conceptualization. JiyZ contributed to conceptualization, methodology, and supervision. MC contributed to conceptualization, writing, and supervision. All authors have read and agreed to the published version of the manuscript.

## Conflict of Interest

The authors declare that the research was conducted in the absence of any commercial or financial relationships that could be construed as a potential conflict of interest.

## Publisher's Note

All claims expressed in this article are solely those of the authors and do not necessarily represent those of their affiliated organizations, or those of the publisher, the editors and the reviewers. Any product that may be evaluated in this article, or claim that may be made by its manufacturer, is not guaranteed or endorsed by the publisher.
